# GenoREC: A Recommendation System for Interactive Genomics Data Visualization

**DOI:** 10.1109/TVCG.2022.3209407

**Published:** 2022-12-21

**Authors:** Aditeya Pandey, Sehi L’Yi, Qianwen Wang, Michelle A. Borkin, Nils Gehlenborg

**Affiliations:** Northeastern University, MA, US.; Harvard Medical School,MA, US.; Harvard Medical School,MA, US.; Northeastern University, MA, US.; Harvard Medical School,MA, US.

**Keywords:** genomics, visualization, recommendation systems, data, tasks

## Abstract

Interpretation of genomics data is critically reliant on the application of a wide range of visualization tools. A large number of visualization techniques for genomics data and different analysis tasks pose a significant challenge for analysts: which visualization technique is most likely to help them generate insights into their data? Since genomics analysts typically have limited training in data visualization, their choices are often based on trial and error or guided by technical details, such as data formats that a specific tool can load. This approach prevents them from making effective visualization choices for the many combinations of data types and analysis questions they encounter in their work. Visualization recommendation systems assist non-experts in creating data visualization by recommending appropriate visualizations based on the data and task characteristics. However, existing visualization recommendation systems are not designed to handle domain-specific problems. To address these challenges, we designed GenoREC, a novel visualization recommendation system for genomics. GenoREC enables genomics analysts to select effective visualizations based on a description of their data and analysis tasks. Here, we present the recommendation model that uses a knowledge-based method for choosing appropriate visualizations and a web application that enables analysts to input their requirements, explore recommended visualizations, and export them for their usage. Furthermore, we present the results of two user studies demonstrating that GenoREC recommends visualizations that are both accepted by domain experts and suited to address the given genomics analysis problem. All supplemental materials are available at https://osf.io/y73pt/.

## 1 Introduction

The almost ubiquitous availability of genomic data has revolutionized research in biology and medicine. By interpreting genomic and epigenomic data, researchers can improve our understanding of the genetic causes and mechanisms that underlie common and rare diseases. Visualization, as an efficient approach for data exploration and knowledge communication, plays a central role in the analysis of genomic data. A large number of visualization techniques and tools have been developed over the years to meet the wide variety of analysis requirements in the field of genomics [38].

To succeed in the visual analysis of genomics data, a genomics analyst or a domain user who may not be trained in data visualization, must carefully select suitable visualizations based on data characteristics and analysis tasks. Currently, genomics analysts use visualizations based on their prior experience or use out-of-the-box visualizations generated by popular genome browsers [19, 24]. In many cases, these visualizations do not consider appropriate visualization best practices, results from empirical studies, or theoretical guidance. For example, a widely used visualization technique in genomics, Circos [23], uses a radial layout with length or position channels that represent quantitative features, which is found to be inaccurate and inefficient in data interpretation [54]. Another example is the lack of effective multi-scale designs (e.g., overviews and detail views) in commonly used genome browser tools as survey results on genomics tools show [27]. These all can result in ineffective and, in many cases, wrong visual design [12, 52]. These and many other limitations of common, as well as specialized visualization approaches in genomics, indicate a need for better visualization guidance.

Given the importance of choosing suitable visualizations, visualization researchers have contributed many visualization recommendation systems, spanning general to domain-specific systems [11, 16, 25, 31, 37, 45, 56, 57]. Existing visualization recommendation systems suggest a data visualization primarily based on the data, and in very few cases, they also consider the user’s tasks [45]. These systems allow data analysts who are not experts in visualization design to focus on the analysis of data and offload the work of visualization design to an algorithm. While these systems have demonstrated success, they are typically designed only for common visualization types, such as bar charts, line charts, and scatter plots, and cannot be applied to scenarios which require complex or domain-specific visual encodings. Genomics visualization is unique and challenging for a number of reasons: First, the design space of genomics visualizations [38] is different from a taxonomy of general visualization [4], including some unique combinations of visualizations that are rarely used in other fields, such as radial overview visualizations and complex glyph encodings for gene structures. Second, analyzing large-scale genomic data requires multiple coordinated views, but existing visualization recommendation systems focus on single-view visualizations. Finally, the analysis tasks in genomics are domain-specific and not considered in other visualization recommendations.

**Fig. 1.**
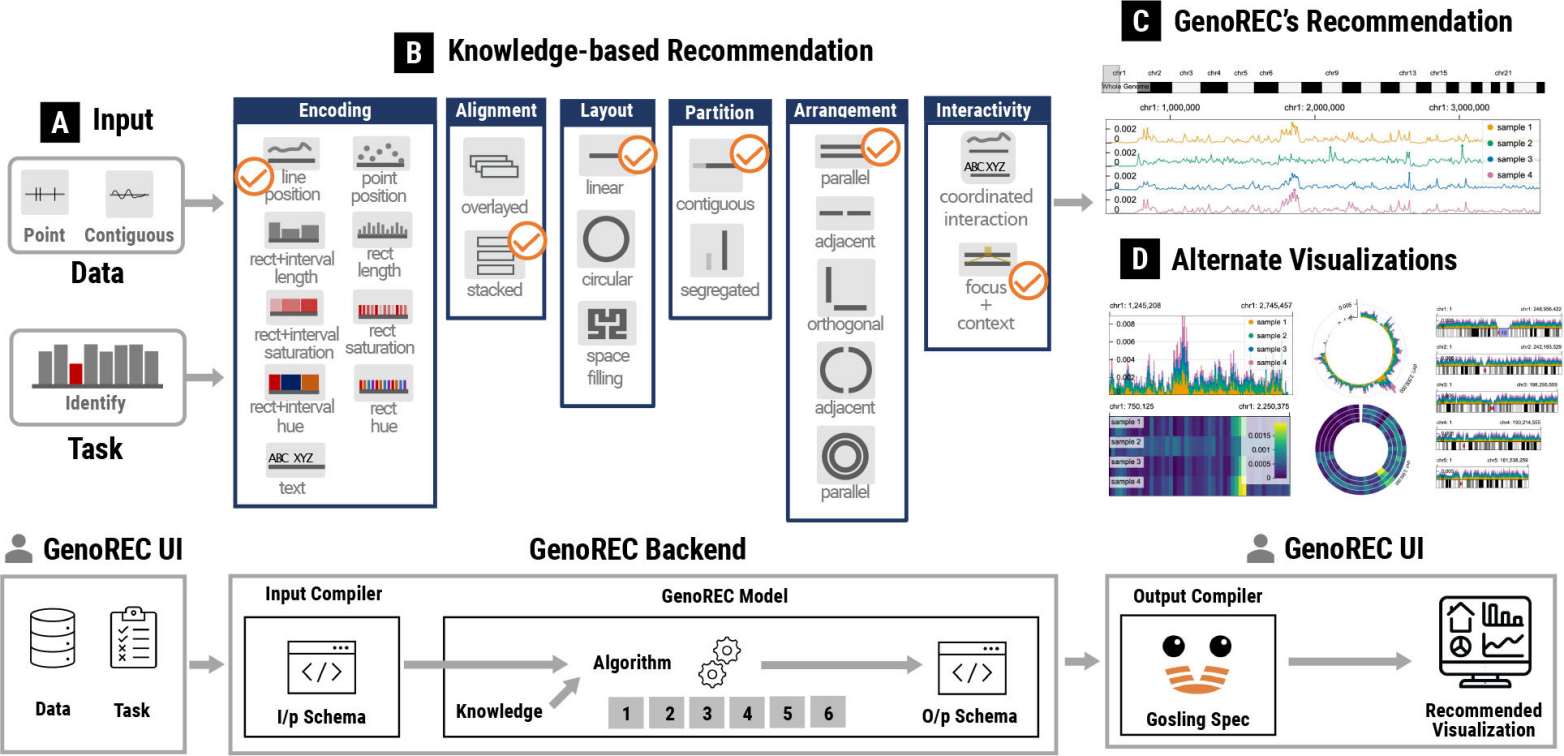
**Top:** GenoREC maps data and task specifications (A) to appropriate visualizations. In this figure, the knowledge-based recommendation (B) shows the component-wise model of GenoREC and the subsequent decisions made at each step. Based on the recommendation model, GenoREC generates and recommends an appropriate visualization to the user (C). Through the recommendation, GenoREC allows the user to avoid a wide range of similar but sub-optimal visualization options (D) given the data and task. **Bottom:** An overview of GenoREC’s system components and their interactions to generate output visualizations.

To address these issues, we designed and developed GenoREC, a novel recommendation system for interactive genomic data visualizations based on specifications about data and tasks. The core contribution of GenoREC is a knowledge-based recommendation model, consisting of a set of decision rules that we summarized based on empirical knowledge and published visualization practices. As shown in Figure 1B, the recommendation of individual visualizations is decomposed into six design components based on the taxonomy proposed by Nusrat et al. [38]. For each component, we enumerate the design space and craft rules to describe the design decisions. In addition to the recommendation model, we contribute a web application that allows genomics data analysts to describe their data and task specifications and explore the corresponding recommended visualizations. The output of GenoREC is a specification for Gosling [29], a grammar-based genomics visualization toolkit. The specification can be used by genomics analysts to customize the recommended visualization for the further use. In this work, we primarily focus on the evaluation of GenoREC’s recommendation model. The results of our user study demonstrate that GenoREC’s recommendations are helpful for analysts working in the space of genomics data analysis.

## 2 Related Work

### Visualization Recommendation System Types:

Kaur and Owonibi [18] found that visualization recommendation systems are designed to take into account four considerations: data characteristics, task orientation, domain knowledge, and user preference. (1) *Data Characteristics* deals with the identification of visual encoding corresponding to the data type of attributes. Mackinlay’s APT system [30] was one of the first systems that implemented automatic mapping from data characteristics to 2D graphics or charts. Polaris (i.e., the research prototype of Tabealu) [50] used APT’s mapping of data characteristics to visual encoding to facilitate the recommendation of visualization. The concept of automatic mapping of data variables to visualization was further extended by the Sage system [44] which included support for more types of visual encoding. More recently, systems like Voyager [56] and Draco [35] were also developed to facilitate data-based visualization recommendations. Draco contributes a technique to learn recommendation knowledge from existing empirical studies. (2) *Task Oriented* recommendations factor in a user’s intentions behind visualizing data as the main criteria for recommending visualizations. The current task-oriented recommendation systems support domain-independent low-level analytical tasks, such as compare and summarize [5, 45, 48]. (3) *Domain Knowledge* imposes further restrictions on the results of the recommendation system as the domain expert may prefer a visualization that is more familiar or widely accepted within their domain. GEViTRec [10] focuses on the domain of epidemiology and recommends a visually coherent combination of charts by inferring a data source graph and mapping that to a set of view templates. (4) *User Preference* relates to factoring end users’ preference in the recommendation system output. For example, Draco [35] has a method to factor user preference in the form of user-defined constraints. GenoREC factors in data, task, and domain-specific guidelines for recommending visualizations.

### Visualization Recommendation Methods:

Visualization recommendation systems can be categorized based on their recommendation method: data-driven and knowledge-based. *Data-driven* systems recommend visualizations for the given input data by learning from a large number of visualization examples. A variety of machine learning models have been employed to learn from a collection of examples, including neural networks [11, 16], reinforcement learning [62], and decision trees [25]. For example, VizML [16] uses a neural network to learn visualization design choices from a large corpus (10^6^) of datasets paired with visualizations. Table2charts [62] employs a reinforcement learning framework to learn step-by-step visualization constructions from a large corpus of tables paired with charts. *Knowledge-based* systems, on the other hand, recommend visualizations by following a set of expert-defined rules and constraints, making the rules more interpretable compared to data-driven approaches [58]. These rules are usually formalized based on the properties of data (e.g., trends or outliers) and the effectiveness of visual encodings [31, 34, 56, 57]. For example, the “Show Me” feature of Tableau [31] provides automatic mark selection based on the data properties type, role, and interpretation. Voyager [56] and Voyager2 [57] rank encodings based on a set of perceptual effectiveness metrics. The multivariate network (MVN) wizard [37] recommends visualizations based on a ranking generated by visualization experts. Beyond the recommendation of visual representations, there is also work on the recommendation of layouts for multi-view visualizations. For example, Kristiansen et al. [22] present an approach that allows semantic alignment of multiple views based on the content of visualizations, such as visual channels used.

Even though data-driven recommendation systems have demonstrated reasonable performance, they usually require many high-quality examples, which are generally not available for domain-specific problems. Knowledge-based recommendation systems can remedy this problem as it is possible to develop a recommendation model by using existing knowledge and best practices from the information visualization field and domain theory. GenoREC uses a knowledge-based visualization recommendation model based on a survey of genomics visualizations [38] combined with design principles in visualization literature [9, 30, 33, 36].

### Genomic Data Visualization Resources:

There are several resources that can assist genomics analysts in choosing an appropriate visualization technique. Nusrat et al. [38] surveyed over a hundred genomics visualization tools and proposed a taxonomy for genomic visualization based on datasets, visual encodings, and tasks. The paper [38] serves as a theoretical resource for analysts to decide an appropriate visualization technique. GenoCAT [13] and awesome-genome-visualization [3] are databases of existing genomic data visualization tools and techniques. They are valuable resources for analysts who want to identify relevant techniques to visualize data. However, these galleries are exploratory and do not prescribe or suggest visualization designs to the users. In the case of genomics, exploratory tools may have limited use because the experts often lack formal training to judge if the suggested options are adequate visualizations. The proposed GenoREC system overcomes this challenge by recommending visualization designs that are accurate from a visualization theory standpoint and appropriate for the given data and task requirements.

## 3 Genome-Mapped Data and Tasks

This section provides an overview of the genome-mapped datasets [53] and tasks with abstractions to support discussions throughout the paper.

### Nomenclature of Genome-Mapped Data:

**Fig. 2.**
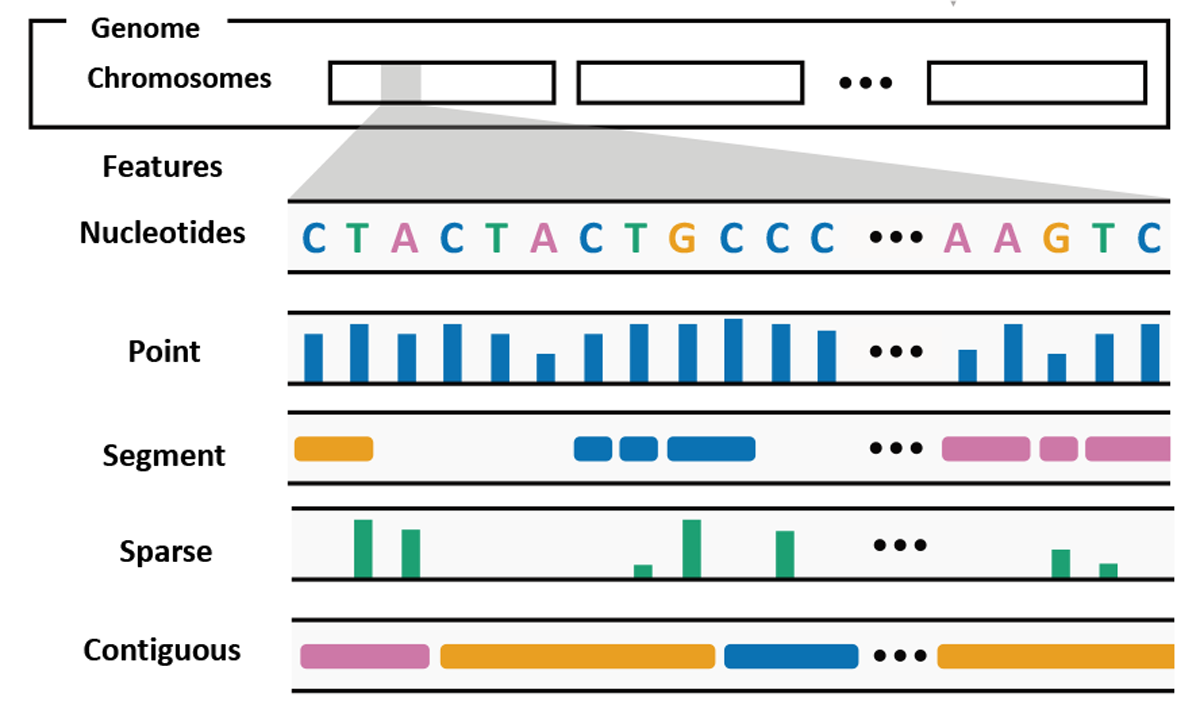
Visual overview of a genome and genomic features: *point*, *segment*, *sparse*, and *contiguous*.

The human genome is a hierarchical structure. As illustrated in Fig. 2, a **genome** consists of one or multiple chromosomes. For functional purposes, chromosomes are subdivided into smaller regions. These regions are called **genes** (feature sets or features). At the lowest level of hierarchy, genomes are composed of building blocks called nucleotides (*A*, *C*, *G*, and *T*). The hierarchical structure makes it possible to measure data at different granularities (extent). Data can be recorded at the individual nucleotide level. This granularity is commonly known as the **point granularity** 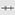 (e.g., single nucleotide polymorphisms, or SNP, that covers only one nucleotide). In other cases, data can be measured at a gene level, which leads to a **segment granularity** dataset 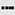 (e.g., genes whose protein-coding regions cover more than one nucleotide). In addition to granularity, the density of information also varies for genomes. In some experiments, data is recorded for each nucleotide, thus leading to a **contiguous data density** 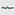 (e.g., DNA conservation scores, which are generally available for each position in of a genome). While in other cases, the data may be **sparse** 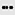 (e.g., genes that only cover a relatively small proportion of the genome), leading to empty and missing values. One of the most distinctive features of genome datasets is that they can also include network-based data and spatially mapped data. Network data in genomes generally represent **connectivity between distant regions of a genome** 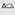 (e.g., physical interactions between different locations in the folded genome), or it may represent **connectivity between two different genomes** 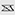 (e.g., synteny [33]). The type of data recorded for genome, also called expression levels, can be three main abstract types: quantitative 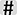, categorical 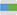 and text 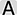. In genomics, textual annotations (e.g., “BRCA2” gene) are important, and therefore they are distinguished from categorical data and are visualized as textual representations. More information on parallels between features in genomics data and other domain data (e.g., geospatial data) can be found in a survey paper [38].

### Visual Analytics Tasks for Genome-Mapped Data:

Genomics researchers perform analytical tasks with data visualizations to explore genomic features and answer critical domain questions. A review of genomics visualization tasks can be found in the survey by Nusrat et al. [38]. Through a thematic analysis of the tasks in the survey using the multi-level task typology of Brehmer & Munzner [6], we categorized genomics analysis tasks into three low-level query tasks. (1) Identify: Analysts are interested in analyzing features in a genomic region of interest to read a specific feature value. For example, navigate to a gene *A* and check its expression level. (2) *Compare*: In this task, analysts want to compare features located in multiple genomic regions. For example, compare the gene expression level between gene *A* and gene *B*. (3) *Overview*: In this task, analysts want to look at a larger genomic region or a whole genome to get an overview of the attributes of a feature set and look for interesting patterns such as outliers, clusters or trends to identify regions for further exploration. For example, analysts want to identify regions with many mutations. The tasks and data introduced in this section play a critical role in design of the GenoREC system.

## 4 Use-Case Scenario

In this section, we present a use-case scenario to illustrate how GenoREC enables analysts to find appropriate visualization recommendations. This scenario is also presented in the supplementary video.

Ada is a genomics researcher working at a cancer research institute. Ada wants to develop a visualization that can help her analyze genetic data obtained from three different files [53]: a BED (Browser Extensible Data) file, a VCF (Variant Call Format) file, and a BIGWIG (Big Wiggle) file. The BED1 file stores the location of genes that are linked with certain diseases. The VCF1 file stores structural variant events of a cancer patient. The BIGWIG1 file, stores protein interactions with DNA. Ada wants to compare the quantitative values of the BIGWIG file between different regions and try to understand if there are specific genetic features in BED1 and VCF1 tracks that are contributing to the measurement of the BIGWIG file.

Ada first tries to visualize the data with conventional genomics visualization tools, such as UCSC Genome Browser [19], IGV Browser [43], and WashU Epigenome Browser [24]. Although these tools can create visuals for specific data types, she is unable to use a single tool to look at all her data and, most importantly, easily combine different file types to perform an integrated analysis. Ada consequently needs to spend a lot of time working across tools and make compromises on her analyses. Moreover, these tools are tightly coupled with the default visualization technique, and Ada would like to explore alternatives and easily customize her visualizations. Finally, the tools do not take Ada’s task into consideration, so there is a great burden on her to be familiar with design guidelines to identify the best encoding. Without guidance on tasks, Ada can choose a misleading visualization [52]. Given these limitations of existing tools, Ada decides to instead try GenoREC to get a recommendation for a visualization that supports her requirements and steers her towards the final plots.

### 1. Describing Data and Exploring Recommendations:

**Fig. 3.**
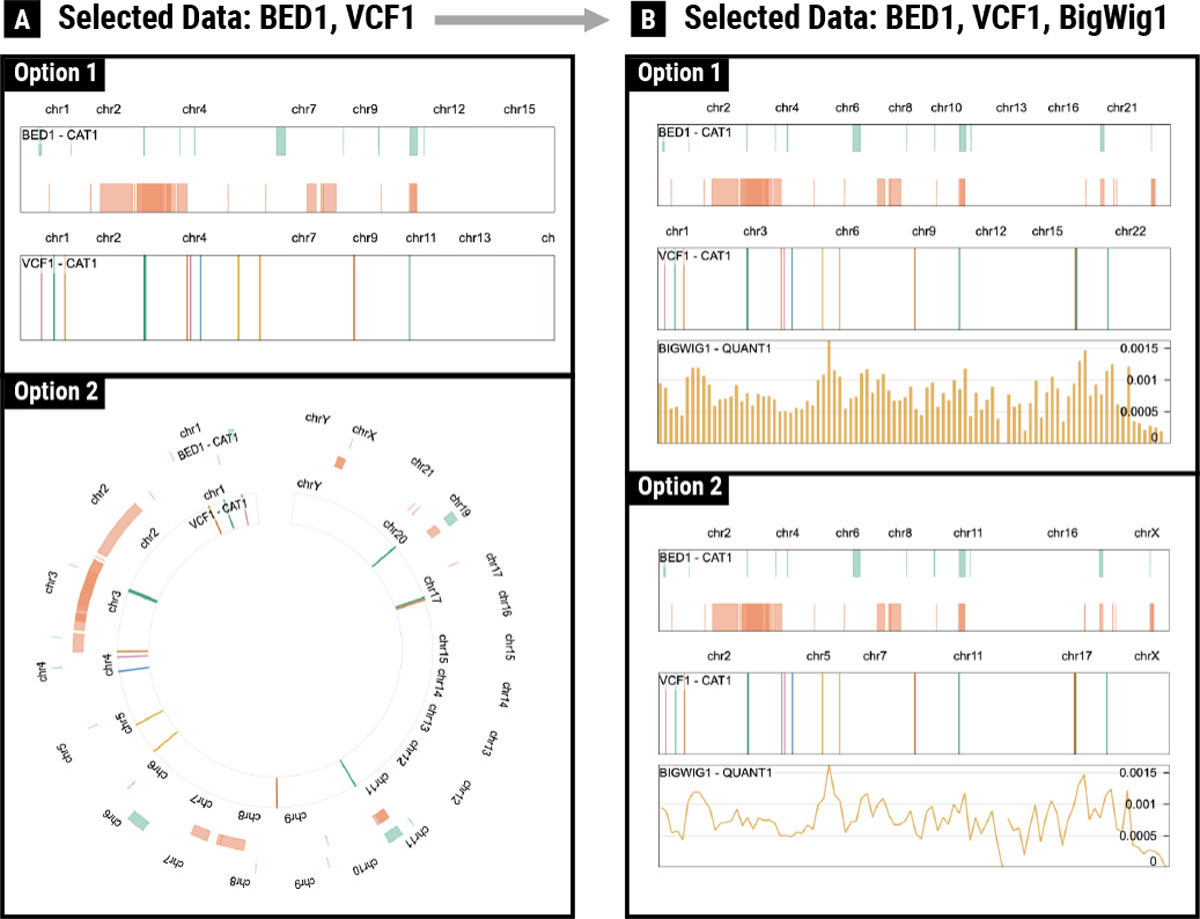
Progression of GenoREC’s recommendation. In *A*, user has specified a BED file and a VCF file with categorical data and GenoREC recommends linear and circular layouts. In *B*, user adds a BIGWIG file with quantitative data and GenoREC switches to a linear recommendation.

**Fig. 4.**
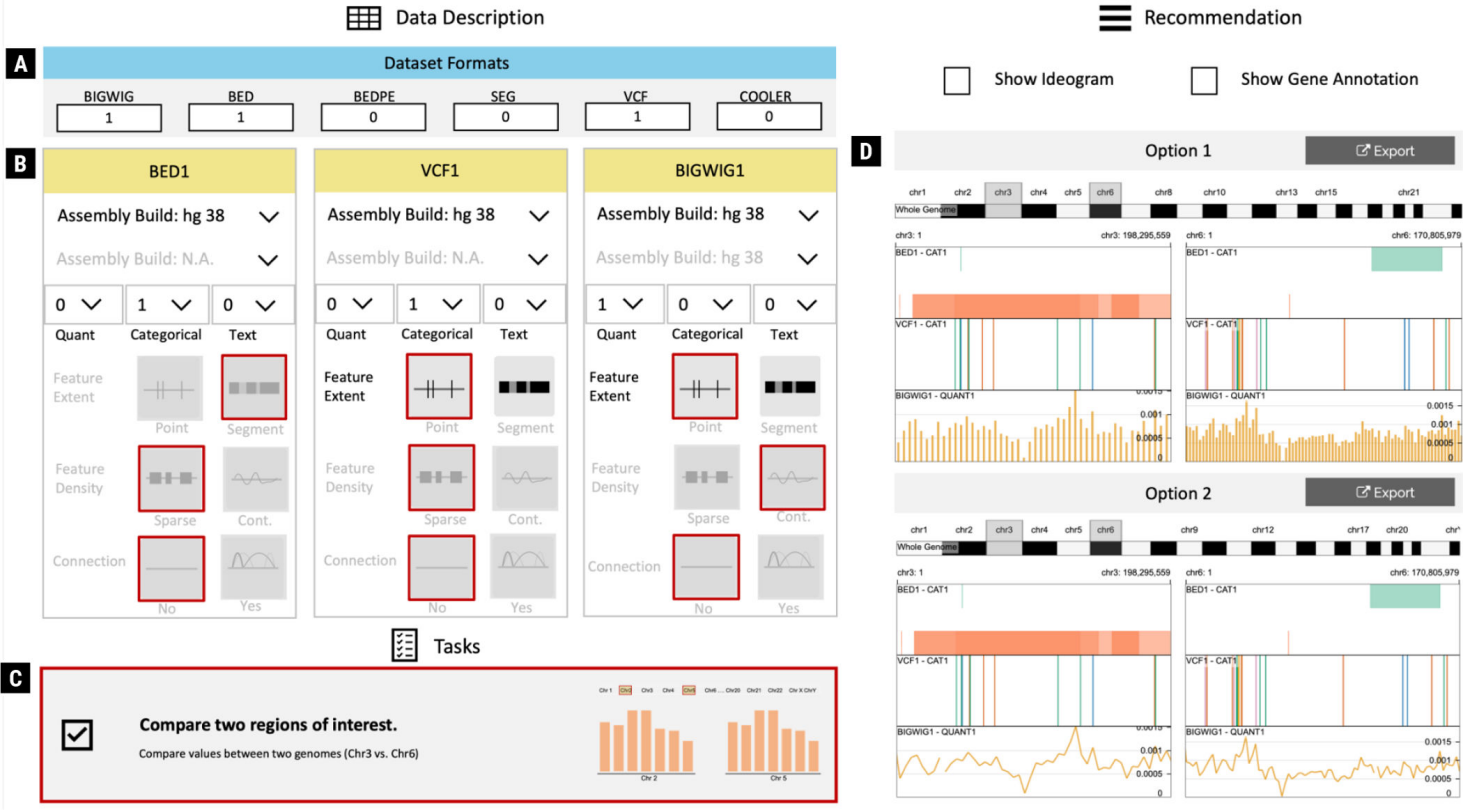
User Interface of GenoREC. The interface consists of two main panels. *Left*: a domain-centric data and task elicitation interface. The data and task specification panel contains two parts data description and tasks. *Right*: a visualization gallery that displays recommends visualizations.

When Ada launches the GenoREC application, she is presented with a data and task specification panel, as shown in Fig. 4 (Left). The data description panel allows Ada to specify the type of data she wants to analyze by using six standard genomics file formats (i.e., BIGWIG, BED, BEDPE, SEG, VCF, and COOLER) [53] (Fig. 4A). Based on her requirements, Ada selects a BED and a VCF file. GenoREC adds two cards (i.e., “BED1” and “VCF1”) to the user interface (Fig. 4B). Next, for each file, Ada configures the input fields based on data characteristics of the input files. For example, for “BED1” file, she selects “1 Categorical.” The remaining input options for the “BED1” file, such as “Feature Extent,” “Feature Density,” and “Connection,” are automatically defined by the system based on the file format. After the selection, Ada notices that the system recommends her two tracks stacked in a linear and circular layout (Fig. 3A). The recommended visualizations allow Ada to see an overview of the genetic features from the two files. These features are visually aligned in the same genomic axis to help her analyze the features concurrently. Next, Ada adds a BIGWIG file description. The updated recommendation removes the circular layout recommendation and shows two linear tracks. The first option uses a bar mark for the quantitative data and the second option uses a line mark for the same data (Fig. 3B). Ada finds both visualizations useful because they support looking up high and low values of the BIGWIG file and analyzing genes in the corresponding BED and VCF tracks.

### 2. Choosing the Task Description:

In addition to data, Ada also chooses her analysis task. GenoREC supports three tasks: “Analyze a Region of Interest in the Genome” (Identify), “Compare Data Between Two Genomic Regions” (Compare), and “Explore the Genomic Build” (Overview) (see Sect. 3). Ada selects the comparison task based on the sample task example shown in Fig. 4C.

### 3. Analyzing the Final Recommendation:

Based on the data and task description of Ada, GenoREC recommends a set of visualization options. Ada also notices that the recommended visualizations are distinct from the previous stage shown in Fig. 3B. The updated recommendation takes into consideration the tasks. Therefore, the genome track is split into two views, and there is a track that supports the selection of a genomic region in the views, as shown in Fig. 4D. Ada appreciates the split view because she can compare two regions to each other without manually navigating between them.

### 4. Exporting and Customizing the Recommendation:

After analyzing the visualization output, Ada decides to export the second option as a Gosling spec. Ada prefers the line chart over the bar chart because it provides a familiar representation of contiguous quantitative data. Ada uses the spec file and customizes the color scale in Gosling [29] and uses this final visualization for further analysis.

## 5 GenoREC Design Goals

GenoREC was developed through an iterative design process. We first analyzed existing literature in recommendation systems and genomics visualization, including genomics visualization tasks (Sect. 3), to identify an initial set of system design goals. Next, we conducted a formative study with five domain experts and solicited feedback from the experts on the recommendation output, user interface, and system workflow (Sect. 7.1). During these studies, we also interviewed the experts to understand their genomics visualization authoring process and current challenges. None of these experts are authors of this paper. Combining the input from experts and using common design suggestions from prior work for general recommender systems [2, 42, 51] and visualization recommendation systems [37, 56, 57], we identified the following goals to guide further development of GenoREC.

### G1 Recommend Domain-Specific Visualizations:

The recommended visualization should be familiar to users in the genomics field. Swearingen and Sinha [51] found that familiar output increases trust within the recommendation model. During the interviews, the experts also emphasized that recommendations should be familiar and easy to understand. Genomics visualizations have unique characteristics that differentiate them from common visualizations outside genomics. For instance, it is a general practice to arrange data attributes available in genomics datasets as parallel tracks. Experts noted that it might require additional effort to understand and communicate the results of an unfamiliar visualization technique or design.

### G2 Use Visualization Best Practices and Domain-Knowledge:

The system should consider best practices from visualization design and the domain to recommend the visualization. A core principle for existing visualization recommendation systems is mapping the data and, in some cases, task requirements to effective visualization designs [35, 37, 45, 56]. Therefore, the system should leverage the knowledge generated by the visualization community through empirical and theoretical research to build rules that guide the recommendation of visualizations. In addition to the general visualization guidelines, the system should also consider domain-specific knowledge for the recommendation. For example, heatmaps are common in genomics [20, 41, 43], but, it is known that the color channel is not optimal for displaying quantitative values [9]. Therefore, the system should ensure that domain practices are not eliminated from the recommendation.

### G3 Support Common File Formats and Tasks for Recommendation:

The system should support common file formats and tasks that are used for the analysis of genomics data. We discuss the BED, VCF and BIGWIG file formats in Sect. 4. Experts noted that the ability to specify data requirements in file formats they are familiar with could reduce the learning curve associated with the system. Therefore, the system should support file formats that support the common data types and structure available in genomics.

### G4 Encourage Fine-Tuning and Iteration of the Recommendation:

The recommendation system should enable users to steer the recommendation progressively. Users may come to a recommendation system when they are not sure what they want, and they need assistance to find an item of interest [42]. Therefore, they may need methods to interact with recommendation inputs in a flexible way and update them until they find an item of interest. To support flexible updates, we also need the interface to support a quick turnaround time in showing the recommended outputs. Therefore, the design of the system should take into account that users will change between different data and task inputs and analyze the change in the visualization recommendation output based on the inputs [57].

### G5 Recommend Design Variations:

Alternative designs of the same data should be recommended to analysts. The design space of genomics visualization is large, and often users may only have familiarity with a small subset of visualizations within the larger design space. Therefore, we see it as an opportunity for the system to recommend design alternatives to users whenever it is possible. Options can improve the trust in the system and may also lead to serendipitous discoveries, which may also lead to a positive attitude towards a recommendation system [21].

## 6 GenoREC

We contribute GenoREC, a novel visualization recommendation system for the analysis of genomics data. A summary of the GenoREC system design and overview of the recommendation workflow is presented in Fig. 1 (Bottom). GenoREC consists of a front-end user interface which allows users to specify their input requirements to the system and browse the recommended visualizations. The system also contains a back-end recommendation model that generates visualization recommendations (“GenoREC Model”). The back-end recommendation model of the GenoREC system generates design configurations for appropriate visualizations given the user input. The two compiler modules: “Input Compiler” and “Output Compiler” of the system are responsible for orchestrating the exchange of information between the front-end and back-end. A key design aspect of GenoREC is its **modular architecture**. This ensures that individual components are easy to update without affecting other system modules.

GenoREC’s user interface is implemented in JavaScript, HTML, and CSS. The back-end recommendation model is published as a standalone JavaScript library on NPM. For rendering recommendation, GenoREC uses Gosling, a grammar-based visualization toolkit for genomics [29].

### 6.1 GenoREC’s Recommendation Model

GenoREC’s knowledge-based recommendation model [2] recommends domain-specific visualizations (**G1**) based on visualization theory and best practices from the genomics domain (**G2**). The model is built on guidelines which map domain-specific input requirements to an appropriate output visualization. In this section, we first present the input and output space of GenoREC’s model. Next, we present the recommendation knowledge used by GenoREC. Finally, we describe the algorithm which allows the system to use the recommendation knowledge to generate the visualizations.

#### 6.1.1 Recommendation Input and Output Space

##### Input:

The data input into GenoREC Fig. 1A is divided into four categories: the assembly build, data type (quantitative, categorical, and text), feature set (feature extent and density), and connection. In addition, the recommendation model also expects the tasks (identify, compare, overview) as inputs. The data and task input are discussed in Sect. 3.

##### Output:

GenoREC’s model breaks down the recommendation task into six intermediate steps: Encoding, Alignment, Layout, Partition, Arrangement, and Interactivity. Each of these six components contains a set of possible output options as shown in Fig. 1B. One of the main contributions of GenoREC is the use of sequential order to better reflect the dependencies between these components. To determine the current order of components, we reviewed the genomics visualization taxonomy [38]. For each part of the taxonomy, we determined precursor steps. For example, we noticed that it was not possible to recommend an orthogonal 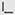 “Arrangement”, if the “Layout” of the tracks were circular 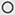. This exercise allowed us to develop the sequence and led to the creation of the sequential model. The sequential order of components ensures that the GenoREC system can accurately recommend visualizations. Here, we provide an overview of each component in the order GenoREC’s recommendation model determines them:

#### C1: Encoding:

The encoding component identifies the visual *mark* and *channel* pair to encode attributes in a genomics dataset. In genomics visualizations, there are four visual marks: *point*, *line*, *rectangle* (“rect”), and *text*. There are also four channels: *position*, *length*, *saturation*, and *hue* [36]. The full list of combinations of marks and channels supported in GenoREC are visually depicted in Fig. 1B.

#### C2: Alignment:

The alignment component identifies if the encodings from the Encoding component (C1) can be *stacked* 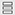 or *overlayed* 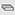. Stacked alignment applies encodings to individual tracks and then vertically stacks them. Here, a track refers to a unit visualization that corresponds to common visualization types [4]. We use this as the default option since this is the most frequent alignment in genomics visualizations [27]. Fig. 3A shows an example of stacked alignment where BED1 and VCF1 files are shown as two separate tracks that are vertically stacked. The overlayed alignment, on the other hand, merges encodings into a single track.

##### C3: Layout:

This component selects the layout to display a track in the genomics visualization. GenoREC supports three layouts: *linear* 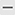, *circular* 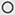, and *space-filling* 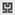
(e.g., the Hilbert curve [14]).

##### C4: Partition:

The partition component decides whether the chromosomes should be displayed in a *contiguous* 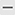 track, where chromosomes are placed end-to-end, or in a *segregated* 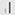 manner, where each chromosome is independently displayed as a separate track. Partition happens at a track level, so if a visualization has multiple tracks, GenoREC determines the division of each track.

##### C5: Arrangement:

This component is responsible for selecting the arrangement of multiple “views”. In this paper, a view refers to a combination of one or multiple tracks. For example, Fig 3B represents two views, each of which consists of two tracks from two data files. The main difference between arrangement and partition is that arrangement is applied between views instead of between tracks. In GenoREC, views that have linear tracks (C3) can be arranged as *parallel* 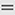, *adjacent* 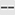, or *orthogonal* 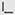. The orthogonal arrangement allows the creation of adjacency matrices for visualizing genomics data with a complete network connection between two sequences. Whereas, if views have circular layout (C3), GenoREC only permits *parallel* 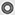 and *adjacent* 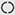 arrangements.

##### C6: Interactivity:

This component is responsible for identifying proper interaction patterns for genomics visualization. GenoREC supports two interaction patterns: *coordinated interaction* and *focus+context*, as shown in Fig. 1B. In the coordinated interaction pattern, all the tracks within a view have coordinated zooming and panning interactions. The focus+context pattern allows users to focus on a specific portion of the genome while maintaining the context of the genome location.

#### 6.1.2 Recommendation Knowledge

This section presents the main design guidelines that shape the recommendation for GenoREC. Additionally, we also discuss how GenoREC uses best practices and domain-knowledge in combination (**G2**).

##### R1: Identify Effective Channels Given the Data and Tasks:

The selection of channels depends on the attribute types, and low-level analytical tasks users want to perform with the visualization. The results of experimental studies by Clevland & McGill [9] and Heer & Bostock [15] led to a ranking of visual channels in terms of their accuracy to identify and compare quantitative data. Based on the results, GenoREC maps quantitative values using position and length channels. Additionally, GenoREC also allows mapping quantitative values of color saturation channel for an overview task. In overview tasks, users are looking for patterns, not individual values, and color saturation can show variation in data for pattern search. For categorical attributes, GenoREC uses categorical color schemes based on the ranking proposed by Mackinlay [30].

##### R2: Choose Alignment Based on File Type and Encoding:

GenoREC supports overlayed alignments for genomics visualizations with multiple tracks only if the tracks are from the same file. They must be from the same file because overlaying tracks from different files can lead to occlusion due to the superposition of multiple marks at the same location [28]. Even if the tracks are from the same file, GenoREC only supports overlaying position and color channels because they can co-exist without causing occlusion. Based on this rule, if a visualization has two tracks with position channels and one track with a color channel, GenoREC will combine the first position channel with the color channel, which will lead to stacked and overlayed tracks.

##### R3: Select Layout Based on Encoding and Tasks:

In GenoREC, layouts are selected based on the input data and task descriptions, and the visual encoding set in the encoding component. In a comparative study with both linear and circular layouts, Waldner et al. [54] found that length and position channels make it easier to identify and compare visual representations in linear layouts. This study recommends linear layouts when length and position are used in the encoding component for identifying and comparing tasks. In overview tasks, where the user’s goal is to look for trends and patterns, GenoREC recommends both circular layouts and linear layouts.

##### R4: Decide Arrangement Based on Layout and Inter-Connectivity Data:

For inter-connectivity data, GenoREC suggests parallel or orthogonal arrangements in linear layouts. Selection between parallel and orthogonal arrangements depends on the type of network data. For dense networks, GenoREC recommends orthogonal arrangements, and in other cases, GenoREC recommends parallel arrangements. For circular layouts, GenoREC recommends adjacent arrangements when there is a network connection, which is one of the most common use cases with circular layouts in the domain [23, 33]. Meyer et al. [33] used a circular adjacent layout for visualizing interconnection between different parts of a sequence. The adjacent circular arrangement allows the analyst to look at overall connections between sequences in a space-efficient manner.

GenoREC gives preference to empirically backed design guidelines. However, when there is a lack of empirical results, GenoREC factors the genomics-specific knowledge for the recommendation (**G2.**). For example, GenoREC recommends color saturation (R1) encoding used for commonly used heatmap visualizations [20, 41, 43], for overview task because there is no conclusive evidence that negates the use of color saturation encoding for overview.

#### 6.1.3 Recommendation Algorithm

##### Representation of Recommendation Knowledge:

**Fig. 5.**
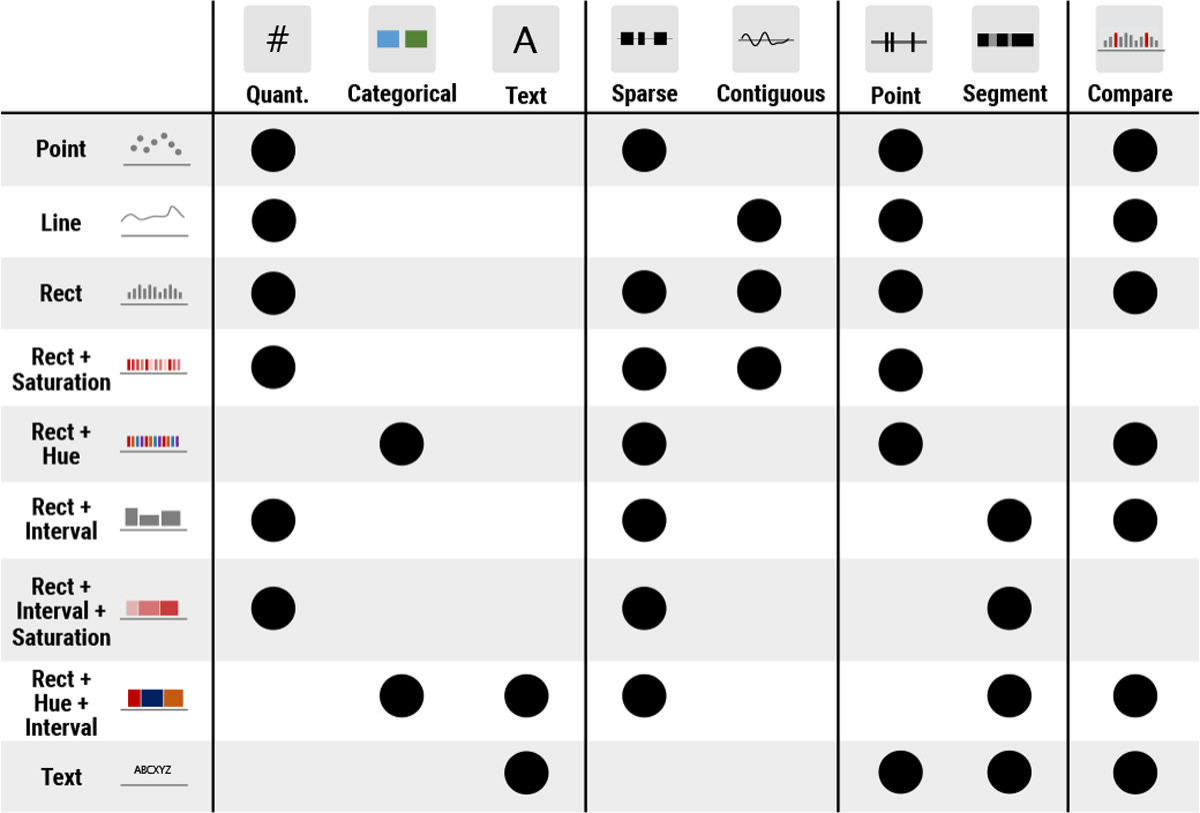
Decision Matrix for the Encoding component (C1). Rows represent the visual encodings, and the columns represent the factors that affect the encoding selection. A 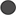 cell in the matrix represents a value 1, which means the encoding supports the factor, and an empty cell represents a value −1, which means the factor is not supported.

The knowledge for recommendation is represented and stored as a decision matrix [47] in GenoREC’s model. For each component (C1–C6) in Sect. 6.1.1 there is a distinct decision matrix. In Fig. 5, we show the decision matrix for the Encoding component (C1). The rows in the matrix represent all the possible output options. The columns in the matrix represent input factors that affect the output selection. Finally, the cells in the matrix represent if the corresponding output (row) can support the corresponding input item (column). GenoREC encodes the relationship between input and output as a binary value. A cell with a dark circle (i.e., 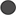) represents a value 1 which means the output is supported by the corresponding input column, and vice versa. For example, in the “Quant.” column (Fig. 5), all the cells corresponding to position (e.g., “Point”), length (e.g., “Rect”), and saturation (i.e., “Saturation”) have a value of 1. However, since the hue channel is not used to represent quantitative data, all cells corresponding to the hue channel have a value of −1 (i.e., empty cells). Representing recommendation knowledge as binary-valued features allows GenoREC to use a similarity computation technique to rank the output of each component (C1–C6) and use the ranking for recommendation.

##### Similarity Computation:

GenoREC computes the similarity between the input and all the output items in the product matrix using a **cosine similarity** metric [8]. The cosine similarity score expects two vectors of equal size and generates a score in the range of [−1,1]. GenoREC’s algorithm generates an input vector for each recommendation component based on the features given in the column headers of the decision matrix. Next, it scores the similarity of this input vector with all rows of the decision matrix. A higher cosine similarity score closer to 1 means that the two vectors are more similar while a lower score closer to −1 means the two vectors are dissimilar. These scores are used for ranking in our recommendation system, i.e., GenoREC recommends the output item with the highest score.

##### Recommendation walk through:

**Fig. 6.**
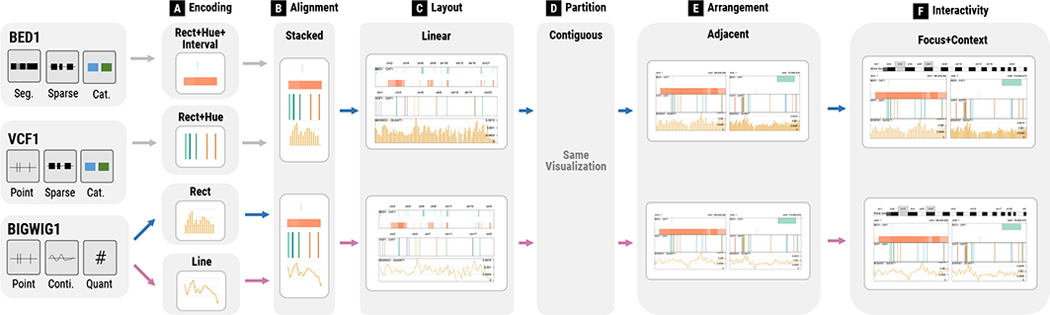
GenoREC’s recommendation creation process: Each component in the model identifies the most suitable output option based on the previous stage and the underlying recommendation model. Output for components (A–F) are explained in Sect. 6.1.3 (Recommendation walk through).

In Fig. 6, we show the steps in GenoREC’s recommendation algorithm with the help of data and analysis task discussed in Sect. 4.

##### C1: Encoding:

GenoREC visually encodes each data attribute in the input data. For example, in Fig. 6A, GenoREC recommends “Rect + Interval” with a hue channel for the “BED1” file because the feature set is a segment and the data attribute is categorical. GenoREC assigns a consistent colorblind-safe color palette for each categorical attribute. For the “BIGWIG1” file, GenoREC recommends both a bar chart (i.e., “Rect”) and a line chart (i.e., “Line”) based on the decision matrix since the file stores point-based contiguous quantitative values (i.e., “Point,” “Contiguous,” and “Quant.”) (Fig. 5). The recommendation of multiple outputs at each component allows GenoREC to recommend design variations to satisfy the design goal (**G5**). After the recommendation, GenoREC uses Cartesian product to determine all the possible output options using the top scored recommendation.

##### C2: Alignment:

GenoREC uses the visual encoding and file type information to determine the alignment of the encodings. Due to the cartesian product, this component has two sets of visualization options in Fig. 6B. In this example, all the visual encodings come from different sources and have different features. Therefore, GenoREC recommends a “Stacked” alignment of visual encodings. The stacking order of visual encoding is consistent to the order of selected files. Fig. 6 illustrates a case where the user selects the “BED” file first and then “VCF,” followed by “BIGWIG”. Therefore, the BED file is placed on the top and others are below it. All the tracks in this component are aligned automatically based on reference genomes.

##### C3: Layout:

GenoREC determines the layout based on the encoding, alignment, and tasks. The space-filling (e.g., the Hilbert curve [14]) layout can only show a single track. The circular layout is not conducive for comparing quantitative data in the BIGWIG file [54]. Therefore, GenoREC recommends only the linear layout (Fig. 6C).

##### C4: Partition:

GenoREC determines the partition primarily based on the user’s task. In this example, to facilitate comparison, GenoREC recommends the “Contiguous” partition, which means that chromosomes are positioned end-to-end in the visualization.

##### C5: Arrangement:

GenoREC identifies arrangement based on the existence of network data, layout (C3), and tasks. In the current example, GenoREC’s decision is based on the comparison task. A single-view visualization can be difficult to compare two regions in the genomes, especially if they are distant, e.g., Chromosome 1 and Chromosome 18. To reduce the need for manual panning and zooming interaction, GenoREC recommends two windows to compare any two regions within the genome.

##### C6: Interactivity:

GenoREC currently recommends interaction based on the visualization arrangement and tasks. In this example, GenoREC suggests “Focus+Context” interaction because it allows users to see a focused region in the genome, which is vital for local comparison tasks.

### 6.2 GenoREC User Interface

GenoREC’s user interface is divided into two panels: data and task descriptions (Fig.4 Left) and recommendation (Fig. 4 Right).

#### Data Description and Tasks:

In line with the domain goal **G3**, GenoREC’s UI allows users to specify domain-specific data (BIGWIG, BED, BEDPE, SEG, VCF, and COOLER) [1, 53] and tasks [38] to the system. The data and tasks are introduced and discussed in Sect. 3. Each data description card (Fig. 4B) in GenoREC has six input fields: Assembly Build (coordinate system 1), Assembly Build (coordinate system 2), Quantitative, Categorical, Text (number and type of data attributes), Feature Extent (point or segment feature set), Feature Density (sparse or contiguous feature set), and Connection (the connection between sequences). GenoREC’s abstract data description technique provides a flexible way for users to configure their data input. Through this interface, analysts can try many different combinations of data input and see the recommendations without going through the data collection and processing pipeline. To ensure analysts choose the tasks correctly, GenoREC includes an example task that communicates to the user when they should choose the particular task and a visual description for additional feedback (Fig. 4C).

#### Recommendation:

The recommended visualizations are shown as a gallery in the visualization panel of the GenoREC interface (Fig. 4 Right). The gallery-based interface supports easy comparison of the recommended options [26]. The recommended visualizations are updated when users add or modify the data or task specifications which enables quick inspection of many different visualization options (**G4**). Additionally, users can export a Gosling [29] JSON specification and directly load its online editor to customize the visualization further if desired.

## 7 GenoREC Evaluation

GenoREC was evaluated in a two-phase user study. First, we gathered qualitative feedback from domain experts (i.e., people who have experience in genomics and visualization) to validate the recommendation output and the user interface design. Second, we conducted a quantitative evaluation where we measured the utility of GenoREC’s recommendation given a set of data and analysis task combinations. The Harvard Institutional Review Board determined that these studies did not require research approval under federal regulations. All participants were volunteers, and they were recruited through advertisements on various Slack teams in the computational biology and genomics communities and Twitter. Study materials from both studies, including the stimuli, tasks, and data analysis code, are included in the Supplemental Material and on OSF (https://osf.io/y73pt/).

### 7.1 Formative Study with Domain Experts

In this preliminary qualitative study, we sought to answer the question: “Does the output from the recommendation system match experts’ expectations?” A clear answer to this question is important because it validates the recommendation rules applied by GenoREC, and its subsequent implementation is valid. This feedback helped us refine GenoREC’s design goals (Sect. 5) and improve the user interface and the system’s overall workflow.

#### Participants and Procedure:

For the formative study, we recruited five domain experts. The participants had worked at the intersection of genomics and visualization with experience ranging from 8–20 years. The study was designed as a semi-structured interview and it lasted an hour. In the study, we presented participants with three different data analysis scenarios in the form of a short paragraph of text. For example, “given a set of BED files with human and mouse genome, identify regions where the data is highly conserved”. Participants were asked to describe an appropriate visualization for each scenario. Then, they were asked to use GenoREC and comment on the accuracy of the recommended visualization. As the last part of the study at the end of the interview, participants were encouraged to provide feedback on the user interface.

#### Findings:

All responses were analyzed by open coding and thematic analysis. From this analysis of participants’ responses, we learned that most participants found GenoREC’s recommendations accurate and only required minor modifications for the scenarios included in the study. This feedback led to the export feature in GenoREC’s user interface. One participant expected the visualizations to include additional biological contexts such as gene annotation track and ideogram plots that provide a summary of chromosomes coupled with genomic location information [40]. Therefore, we added an option to add a gene annotation track and ideograms to the visualization in GenoREC. Another participant asked for an additional interactive component as a tabular view in the GenoREC interface that enables a faster lookup of gene location. Since we mainly focused on the recommendation of visual representations in the GenoREC interface, it is not included. However, we plan to add the feature to further enable effective visual exploration of genomics data. Finally, GenoREC’s user interface was well-received, and participants understood the data and task descriptions. One participant stated that it is possible to further abstract the domain-specific file inputs. But, we decided to keep data descriptions in domain-specific terminology because other participants found it helpful.

### 7.2 Quantitative Evaluation with Genomics Analysts

In the second part of the study, executed after feedback from the qualitative study, we conducted a controlled within-subject quantitative study to measure the difference in utility between GenoREC’s top-ranked visualizations and mid-ranked visualizations for a given set of datasets and analysis tasks. While the qualitative study gave us feedback on the accuracy of the recommendation, we still wanted to evaluate if the recommendation would be useful for a broader group of people working in genomics. Since GenoREC’s recommendation model considers best practices from visualization research and domain knowledge based on common data features and tasks, we hypothesized that participants will find GenoREC’s top-ranked recommendation to be more useful than a mid-ranked visualization.

We collected datasets from multiple sources that lead to diverse data descriptions in the study. For contiguous quantitative values, we used multiple samples of ChiP-seq data [61]. For sparse quantitative and nominal values, we used various datasets including GWAS catalog [7], gene annotations [17], and somatic structural variants [49]. We also used the structural variants data to represent connectivity information within genomic locations.

This experimental design was chosen instead of a direct comparison to another recommendation system as there is no other system available for direct comparison. For example, existing generalized recommendation algorithms evaluated by Zeng et al. [60] do not consider domain-specific aspects such as layout and arrangement. The autoplot feature of ggBio [59], which is the closest alternative to GenoREC, does not support interactive visualizations or task-based recommendations.

We developed a separate web application for this study because some features of the GenoREC app, such as the task description (e.g., Fig. 4C), could have revealed the expected output and affected the results. Participants were shown one visualization at a time. To compare responses across tasks and participants, we used a Likert scale-based response format.

#### Participants:

We recruited 13 participants (P1–P13) with three participants who identified as female and ten who identified as male. Anyone who had experience with genomics data analysis was qualified to participate in the study. Participant experience in genomics ranged from 6 months to 18 years, with an average experience of 4 years.

#### Evaluation Scenarios and Stimuli:

There were a total of nine data and task scenarios (Scenario 1–9) in the study. The scenarios are designed to provide a comprehensive coverage of three factors: features, attributes, and tasks. For example, in Scenario 1, the feature combinations are point and sparse with no connection, the attribute used is categorical, and it is an identify task. We ensured that the scenarios cover all feature types (point, segment, contiguous, sparse, and network) and attributes (quantitative, categorical, and text) at least once. Finally, we ensured that tasks were balanced among identify, compare, and overview.

To create the stimuli for the study, we used GenoREC to generate and score all possible visualizations that can be rendered by Gosling for the data and tasks of a given scenario. From that list, we selected the visualization with the top score (“GenoREC stimulus”) and the visualization that is assigned with the score closest to the median of the score distribution (“alternate stimulus”). If GenoREC recommends multiple top and median ranked visualizations with the same score, we randomly select one of them as a stimulus.

#### Procedure:

The study was conducted synchronously online. We arranged a 30-minute evaluation session with each participant via Zoom. The sessions were not recorded. In the first ∼5 minutes of the session, the interviewer asked participants about their experience with genomics data and explained the study setup by demonstrating the web application. After the introduction and demonstration of the application, the interviewer requested the participant to open the web app and share their screen so that the interviewer could follow the progress and answer any questions. Each scenario in the study had a data and task description. Example data and task description for Scenario 1 is the following:

#### Data:

File Format: VCF, Attributes: 1 Categorical, Extent: Point, Density: Sparse, Connection: No

#### Task:

Navigate to the window chr19: 20,000,000–chr19: 80,000,000 and characterize the distribution of the categorical variable, i.e. similar values or distinct values.

**Fig. 7.**
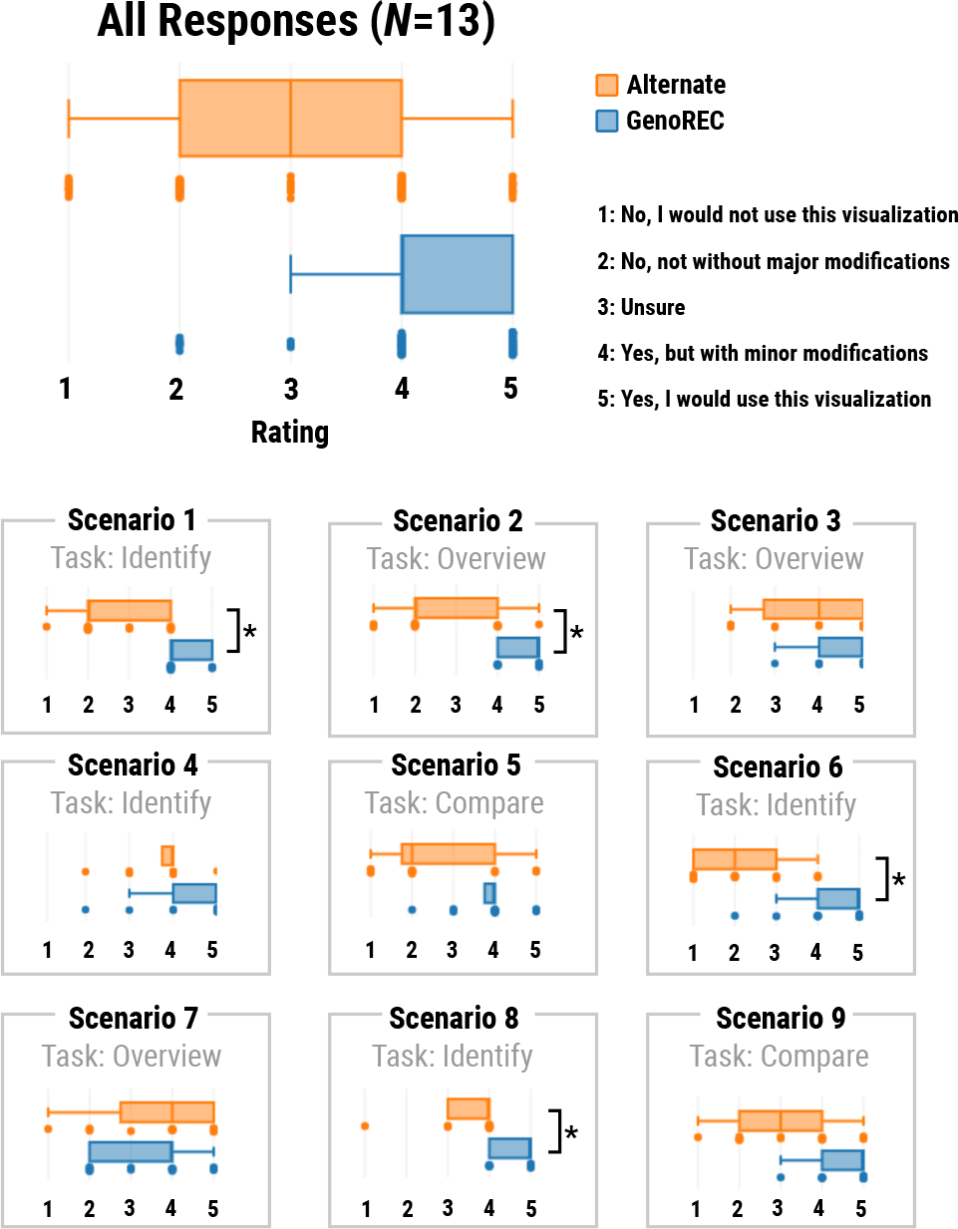
Utility ratings for GenoREC and Alternate stimulus across all participants and scenarios. Scenarios where GenoREC’s responses were significantly higher than the alternate stimulus, are marked with *.

Along with the description, the participant saw one of the two visualization options at a time. In each scenario, the data and task description remained the same for both visualization options. Participants were asked to perform the task with the visualization and then answer the following question: “I would use this visualization for the analysis of the data given the task description.” The responses were recorded on a 1–5 scale as shown in Fig. 7. The stimuli shown in the scenarios were randomized to account for any learning effect. During the study, participants were not required to provide feedback, but many participants followed a think-aloud method where they explained the reason behind their ratings. We recorded this feedback as transcribed notes and used it for further analysis of the responses.

#### Data Analysis and Results:

To summarize participants’ rating responses, we plot the combined and scenario-wise distribution of responses for both GenoREC and alternate stimuli using box plots (Fig. 7). We find that the median score is higher for GenoREC’s combined responses (*median* = 4) than for the alternate stimuli (*median* = 3). Additionally, the interquartile range (*IQR* = 1; *Q*1 = 4, *Q*3 = 5) of GenoREC is less dispersed than the alternate stimuli’s interquartile range (*IQR* = 2; *Q*1 = 2, *Q*3 = 4). The median and interquartile responses for all participants combined are shown in Fig. 7. We conclude that (1) participants found GenoREC’s top recommendation more useful than an alternate stimulus in most cases and (2) participants are less confident with the rating of the alternate stimulus, which led to a more dispersed interquartile range (*IQR* = 2; *Q*1 = 2, *Q*3 = 4). We used the non-parametric the **Wilcoxon signed-rank test** [55] to measure if the scenario-wise median difference in responses varies between GenoREC and alternate stimulus. To handle multiple comparisons, we used a Bonferroni corrected p-value of 0.0055. As illustrated in Fig. 7, for Scenarios 1, 2, 6, and 8, GenoREC’s responses are significantly higher than alternate stimulus responses with *p* < 0.0055 for the Wilcoxon test.

#### Scenarios for which GenoREC’s recommendation performed better:

Scenario 1 (*p* = 0.0018) and Scenario 8 (*p* = 0.0021) have the strongest signal for GenoREC’s stimuli among all the scenarios. We used the qualitative feedback from the participants to understand why did GenoREC perform better than the alternate stimulus? In Scenario 1, we compared a linear layout with a focus+context (GenoREC) to a circular layout (alternate) in an identify task (as described in the “Procedure” description). We learned that some analysts did not like the circular layout from the participant feedback. Multiple participants (P5, P6, P7, and P10) explicitly noted that they do not prefer to use circular layouts. P7 mentioned that they generally avoid using circular layouts for their genomics visualization. An additional reason for preferring GenoREC’s recommendation was the focus+context interaction. The focus+context interaction simplified the task of navigating to a specific genome region. For Scenario 8, GenoREC recommended a visualization stacked alignment, and the alternate recommendation overlayed the tracks into a single view. Participants found the overlayed tracks overwhelming and did not add any benefits to the stacked layout.

#### Scenarios for which GenoREC’s recommendation did not perform better:

For Scenario 3 (*p* = 0.08) and Scenario 7 (*p* = 0.35), we did not find a large difference between the GenoREC and the alternate stimuli. For Scenario 3, we observed that participants found both GenoREC (*median* = 5) and alternate stimulus (*median* = 4) useful. In this scenario, participants were asked to perform an overview task (look for patterns) using a linear track with contiguous partition (GenoREC’s stimulus) and a circular track with segregated partition, i.e., one circle per chromosome (alternate stimulus). P2 mentioned that a circular segregated layout allowed them to see more granular data, which supported them in searching visual patterns. The most surprising results came from scenario 7, where the interquartile range of GenoREC (*IQR* = 2, *Q*1 = 2, *Q*3 = 4) was less than the alternate stimulus (*IQR* = 2, *Q*1 = 3, *Q*3 = 5) based on the summary. In this scenario, GenoREC presented two circular tracks stacked (similar to Fig. 3A Option 2), and participants had to perform a correlation task. We expected participants would use an overview strategy. However, they preferred to interact with the tracks and zoom into specific regions of the circular tracks. As discussed in the feedback from Scenario 1, circular tracks are not effective for tasks in which users have to focus on a local region. Based on this result, we learned that users could solve correlation tasks through an overview or a region-based focus approach. Therefore, GenoREC’s model should provide users with features that allow them to focus on specific regions for correlation tasks.

#### Summary:

The results from the quantitative evaluation show that GenoREC’s recommendations are rated higher than the alternate recommendation. The qualitative feedback indicated that circular layouts do not work well with tasks requiring navigation with zooming and panning. Additionally, users liked the focus+context recommendation because it simplifies navigation to specific regions in the genome. Since our goal was to execute a study of reasonable duration for participation (∼30 minutes), we could not exhaustively evaluate all the possible data and task combinations. A small sample size of 13 participants is a limitation of the study. However, due to the specialized nature of the domain, it is a challenge to conduct the study with a larger number of participants. Finally, since we mainly used subjective responses from participants, additional follow-up studies would be required to validate objective aspects of the recommendation model (e.g., validating actual coverage of the design space). However, given the lack of ground truth data and the unavailability of comparable recommendation systems or algorithms, our evaluation methodology of using subjective responses from domain users presents a practical way of assessing visualization recommendations for genomics.

## 8 Discussion

### Generalizability of GenoREC’s Recommendation Model:

We present a sequential recommendation model for genomics visualization. To develop the sequential model, we broke down visualization techniques in genomics into multiple components (e.g., encoding, layout) and added order to them (Sect. 6.1.1). We believe the sequential method can be applied in other domains and visualization techniques. For example, tree visualizations can be decomposed into three components based on a visualization taxonomy [46]: “Representation” (explicit, implicit, hybrid), “Alignment” (parallel, radial, free), and “Dimensionality” (2D,3D, hybrid). To apply the sequential model, tree visualization experts can determine the dependencies between components, such as whether “Representation” of tree visualizations should be determined before “Dimensionality”. After ordering the components, domain experts can systematically curate recommendation knowledge for each component and create a sequential recommendation model for tree visualizations. However, the sequential model may not apply universally across all domains and visualization techniques. For instance, applying this method to infographics can be challenging because they offer a lot of design freedom that it is challenging to identify its design space. This makes it difficult to decompose visualization into smaller components, restricting the use of the sequential model. Despite its application limitations, we believe using a sequential model can simplify the process of designing a knowledge-based recommendation model for domains and visualization techniques where it is possible to break down visualization into components and apply order to them. Therefore, we anticipate to see it adopted more in the future.

### Updating and Extending GenoREC’s Recommendation Model:

We can update GenoREC’s recommendation model by changing each component’s output items and decision factors. For example, to introduce a new glyph encoding in the Encoding component (C1), we need to add a new row to the decision matrix (Fig. 5). Adding additional decision factors is also trivial and requires the addition of a new column to the decision matrix of each component. For instance, if we want to factor in user preferences for recommended encodings, we can add a new column in the decision matrix, which stores the relationship between encoding output and the user-preference factor. Component-level changes in GenoREC are straightforward as they do not affect other aspects of the model. However, extending the recommendation model to handle a new component could be challenging. Adding a new component requires careful analysis of its dependencies with all the other components, which can be a complex if done post-hoc. Therefore, we recommend that researchers err on the side of inclusion when identifying the components to be used with GenoREC’s recommendation approach.

### Future Work:

Currently, GenoREC’s model is accessible through the user interface. In the future, we plan to package the model into a library that can be accessed programmatically and embedded into tools like Gos [32] that enables the generation of Gosling specifications through Python, e.g., in Jupyter Notebooks. In this context, it will also be possible to generate visualizations based on actual data files rather than descriptions of the files. Furthermore, with the ability to read data, many additional features can be included, such as optimal color scale based on data, or coordinating color scales across similar attributes in separate tracks as recommended by Qu and Hullman [39]. Another interesting direction of future work would be to consider user preference in GenoREC’s recommendation model. Currently, we do not have user preference data for genomics visualizations. However, with the adoption of GenoREC, we can get more information on visualizations that users prefer, which can be used in the recommendation model for ranking the output. Finally, in the future, we intend to explain the recommendation to the users. Through explanation, we would justify the reason behind a recommendation to answer questions the domain users may have, i.e. “Why was a linear layout recommended over a circular layout?”

## 9 Conclusion

Analysis of genomics data continues to be the backbone for many critical biomedical inventions and discoveries. Genomics data analysts heavily rely on visualization techniques for data interpretation, which will be made more efficient through the support provided by GenoREC in constructing appropriate interactive visualizations. Our design and algorithm can be extended to create other domain-specific visualization recommendations, where the visualization and task taxonomy have been well defined. Ultimately, our work offers critical guidance for visualization researchers who want to develop similar recommendation systems in other domains.

## Supplementary Material

Supplementary Material

Supplementary Video
