# Supplementary material for: GenoREC: A Recommendation System for Interactive Genomics Data Visualization
Source: IEEE Trans Vis Comput Graph. Author manuscript; Available in PMC 2023 Apr 5. (PMC10067538; doi:10.1109/TVCG.2022.3209407)
Supplement: Supplementary Material [file NIHMS1846026-supplement-Supplementary_Material.zip › Supplemental Materials GenoREC/Supplemental Material Readme.docx]

Supplemental Material

GenoREC: A Recommendation System for

Interactive Genomics Data Visualization

This document is a lookup reference for the items included in support for the GenoREC paper.

1. **Folder: Study 1**
   1. *File: GenoREC Study1:* This file includes information for the study 1 with domain experts. This file was used to anchor the conversation. Therefore, it includes detailed step by step information of how the study was conducted. It also includes the questions we asked the participants and the tasks they performed during the study.
   2. *File: Appendix for Study 1:* This document summarizes the study procedure in detail and includes a lengthy description of our findings from the study.
2. **Folder: Study 2**
   1. *File: GenoREC Study 2:* Similar to previous folder, this file includes step by step procedure of how participants were oriented for the study.
   2. ***File: Data_tasks_stimuli.pdf:* This file includes Scenario 1-9 used in the study and described in the paper**
   3. *Folder: Anonymized Participant Responses:* This folder includes a zip file that includes all the responses from the participants
   4. *Folder: Data Analysis Code:* This file includes python notebooks used for processing and analyzing responses from the participants and performing statistical tests.
   5. *Folder: Final Processed Data:* The final data that was created for visualization and statistical tests.
   6. *File: Appendix for Study 2:* This file provides additional details for stu
3. **File: Cover Letter of GenoREC 2022.pdf:** This file is a copy of a file uploaded to PCS. The file highlights the changes we have done since the last submission.
