# Supplementary material for: GenoREC: A Recommendation System for Interactive Genomics Data Visualization
Source: IEEE Trans Vis Comput Graph. Author manuscript; Available in PMC 2023 Apr 5. (PMC10067538; doi:10.1109/TVCG.2022.3209407)
Supplement: Supplementary Material [file NIHMS1846026-supplement-Supplementary_Material.zip › Supplemental Materials GenoREC/Study 1/Appendix for Study 1.docx]

### Appendix for Study 1

**User-centric Evaluation**

In order to evaluate GenoREC, we conducted a user study with genomics analysts. In our user-centric study, we gathered feedback from domain users about the perceived recommendation accuracy of the GenoREC. Among many other factors, user-centric evaluation can gather data on the system's accuracy, design and usability of the recommendation interface, novelty and familiarity of the recommended output, and interpretability of recommendation output.

Through out study, we sought to answer the following question: "Does the output from the recommendation system match the expectations of analysts?" We were particularly interested in the feedback of the genomics analysts because the system was designed to support them find appropriate genomics visualizations. We used a semi-structured interview to gather users' opinions about the accuracy. Additionally, our study included questions about user interface design, familiarity of the recommendation output, and whether recommendation of alternatives is desirable. Factors like usability of interface and familiarity with recommendation output can significantly improve the experience of the user with the system. We did not conduct a quantitative evaluation because there is currently no ground-truth data on what constitutes a "correct'' genomics visualization. This study was reviewed and approved by Harvard University's IRB.

**Participants**: For our study, we recruited five genomics analysts: two analysts were affiliated with Harvard University, one affiliated with Dana-Farber Cancer Institute, one affiliated with MIT, and one affiliated with a biotech company. All of the analysts have a PhD in computational biology or computer science. The range of analysts' experience with genomics data analysis varied from 8 - 20 years. All participants had previously used genomics visualization tools, while two have also developed tools to visualize genomics data.The participants were recruited through advertisement on Harvard University's internal message board and through email. Participants volunteered their time for the study and were not compensated in any form.

**Procedure**: The qualitative study was conducted synchronously online. We arranged an hour-long evaluation session with each participant via Zoom. The sessions were not recorded. In the first ~10 minutes of the session we asked participants about their professional role, experience with genomics data, and visualizations to ensure we analyze their response with proper context. After the introduction, using a reproducible script we spent ~10 minutes on the demonstration and explanation of the user interface. After the demonstration, we asked participants about their feedback on the user interface. The participants were encouraged to discuss anything they found confusing. The scenarios and associated data and task are included in the Supplemental Materials. For each scenario, participants were asked to verbally describe visualizations they thought would be suitable for the given scenario. After participants described the visualization, they entered the same data and task specifications in GenoREC and commented on the accuracy and familiarity of the recommended visualizations. In cases where GenoREC showed design variations, we also asked the participant's opinion on the variations. These three tasks lasted~30 minutes. We used controlled scenarios so that we could compare results across participants. The final ~10 minutes of the study were allocated for free-form exploration of the system. In the last 10 minutes, participants were free to provide data and task scenarios of their choice, and we asked them the same follow-up questions that we asked them in controlled scenarios. At the conclusion of the study session participants were encouraged to provide any general feedback on the system.

### Findings and Insights

**Perceived Accuracy of Recommendation**:Three out of five participants (P1 - P3) found that for all three data analysis scenarios GenoREC recommended visualizations that matched their expectations. There was some degree of variation in the confidence of the responses. Participants used the phrases like "pretty much what I expected," indicating that the general idea was similar. In some other cases, we got more confident responses "Yeah!". We did not find any particular correlation between the confidence of reactions and the scenarios. The degree of variation indicated that participants wanted more specific customization in the visualization. For instance, in one scenario, the task was to explore the entire genome and identify peaks and dips. However, a participant (P3) mentioned it would be good if the visualization highlighted the peaks. Responses from one participant were mixed (P4). They found only one recommendations accurate. Their main concern was related to the fact that the data did not match the biological context. Their responses focused on the data shown in the visualization rather than on the visualization design. One participant (P5) found that all the recommendations were different from their expectations. They pointed out the recommendation system needs to include tabular views for representing regulatory regions data in addition to the current visualizations. We made interesting observations when participants were allowed to provide their input. At that point they all saw potential in the system. P4, who had mixed responses at first, was excited to see the potential recommendation space and commented "This is cool!" and said the tool has "potential".

**Design and Usability of the User Interface:** During the user interface demonstration, we observed the things that confused participants. Generally, we found the user interface was well-received, and participants understood the data and task descriptions. Moreover, no participant expressed concerns about the fact that they cannot upload their own datasets. All participants understood, and some supported the design decision. One participant (P2) mentioned that they have worked on parsing of genomics datasets. They highlighted that it is a complex problem and argued that we should not even use the concept of files, instead use a more abstract data specification input interface.

**Familiarity and Diversity of Recommendation:** All of the participants found the recommended visualizations familiar. Two participants (P1 and P5) found it useful to see alternatives because it gave them more design visualization options. Other participants found that the design variations could be potentially useful, but they said they would prefer the one they are familiar with and commonly use.
