# Supplementary material for: GenoREC: A Recommendation System for Interactive Genomics Data Visualization
Source: IEEE Trans Vis Comput Graph. Author manuscript; Available in PMC 2023 Apr 5. (PMC10067538; doi:10.1109/TVCG.2022.3209407)
Supplement: Supplementary Material [file NIHMS1846026-supplement-Supplementary_Material.zip › Supplemental Materials GenoREC/Study 1/GenoRec Study 1.pdf]

GenoRec Study with Domain Experts

Evaluators: Aditeya Pandey and Sehi L'Yi

Affiliation: Harvard Medical School

Information Sheet:
https://docs.google.com/document/d/1CBofrsyecQvLtAiywFumlDX72ConEybbQDDlVTcuP3k/edit#

GenoRec

GenoRec recommends genomic visualization designs based on data characteristics and analytical tasks.

In the current version of the system, we do not support users to upload their genomic data files. Instead, we allow users to describe the characteristics of their datasets they want to analyze and use these descriptions to generate the recommendation.

Applications: GenoRec recommendations can serve as a starting point for genomic analysts to enhance their visualizations or as a final visualization design for analyzing genomics data.

Study Procedure

1. Participant Information and Background - 10 mins
2. First, we will give you a tutorial of the interface. - 10 mins
3. Next, we will show you three recommendation tasks for three dataset characteristics, they will also contain task descriptions. - 30 mins
    a. For each recommendation task, we will ask you some follow up questions to understand the validity and usefulness of the recommended output.
4. After, the recommendation tasks, you can either use the system and try it out for with your own data and task descriptions, or we can get that information from you and generate the output. If you are comfortable please share your screen. We will ask you questions about recommendation output. - 5 mins
5. Finally, we will ask for your feedback with the user interface and overall experience with the system. - 5 mins

Participant Information

1. How many years have you been working in the field?
2. Can you please tell us about your professional role and work experience?
3. Have you previously worked on genomics data analysis? What kind of data and analysis have you worked with?

Experience with Genomics Visualization Tools

1. Can you name a few genomics visualization types or tools that you have used?
2. What are the tools that you have used?
3. What do you like about these tools?
4. What are the things that you feel can be improved in these tools?

User Interface Tutorial

1. Data Description Cards
2. Task Description Cards
3. Show Recommendation Button

Recommendation Task 1

Data Characteristic

You are given a set of DNA methylation profiles in 4 BIGWIG files and a genomic region of interest.

Task Characteristic

Check if the DNA methylation profiles are similar in the 4 BIGWIG files within the region of interest.

Can you suggest a visualization?

Quality of Recommendation

1. Did the recommended items match your expected outcome?
2. (Which items that matched your expectations and which ones did not?) - Pointers for more discussion.

3. Have you seen these visualizations before? (Familiarity and Novelty)
4. (Which items were familiar and which ones were novel?)

5. Do you think the system recommended you a diverse range of visualizations?
6. (Can you explain your response?)

Recommendation Task 2

Data Characteristic

You are given a set of regulatory regions in human in a BED file along with conservation scores for human and mouse genomes in BIGWIG files mapped to human genome coordinates.

Task Characteristic

Identify regulatory regions that are highly conserved in both human and mouse.

Can you suggest a visualization?

Quality of Recommendation

1. Did the recommended items match your expected outcome?
2. (Which items that matched your expectations and which ones did not?) - Pointers for more discussion.

3. Have you seen these visualizations before? (Familiarity and Novelty)
4. (Which items were familiar and which ones were novel?)

5. Do you think the system recommended you a diverse range of visualizations?
6. (Can you explain your response?)

Recommendation Task 3

Data Characteristic

You're given copy number data in a SEG file, genomic variants in a VCF file, DNA methylation data in a BIGWIG file and a collection of genomic regions in a BED file.

Task Characteristic

Describe the information for one of the genomic regions contained in the BED file.

Can you suggest a visualization?

Quality of Recommendation

1. Did the recommended items match your expected outcome?
2. (Which items that matched your expectations and which ones did not?) - Pointers for more discussion.

3. Have you seen these visualizations before? (Familiarity and Novelty)
4. (Which items were familiar and which ones were novel?)

5. Do you think the system recommended you a diverse range of visualizations?
6. (Can you explain your response?)

Recommendation Task 4

Data Characteristic

You are given 5 BIGWIG files containing the containing a signal for histone modifications.

Task Characteristic

Compare the strength of the signal (height of the peaks) across two genes of interest to determine which for which gene the signal for each of the 5 histone modifications.

Can you suggest a visualization?

Quality of Recommendation

1. The items recommended to me matched the outcomes that I was expecting. Agree or Disagree?
2. Which items that matched your expectations and which ones did not?

3. The items recommended to me were familiar. Agree or Disagree?
4. Which items were familiar and which ones were novel?

5. The items recommended to me were diverse. Agree or Disagree?
6. Can you explain your response?
