# Supplementary material for: GenoREC: A Recommendation System for Interactive Genomics Data Visualization
Source: IEEE Trans Vis Comput Graph. Author manuscript; Available in PMC 2023 Apr 5. (PMC10067538; doi:10.1109/TVCG.2022.3209407)
Supplement: Supplementary Material [file NIHMS1846026-supplement-Supplementary_Material.zip › Supplemental Materials GenoREC/Study 2/Data_tasks_stimuli.pdf]

Data, Tasks and Stimuli for Quantitative Evaluation (Study 2) – Page 1/3

Study Web App: https://aditeyapandey.github.io/genorec-study/

Scenario	Data	Task	GenoREC	Alternate
1	#1<br>File Format: VCF<br>Attributes: 1 Categorical<br>Extent: Point<br>Density: Sparse<br>Connection: No	Task: Navigate to the window: Chr19: 20,000,000 - Chr19: 80,000,000 and characterize the distribution of the categorical variable i.e. similar values or distinct values.	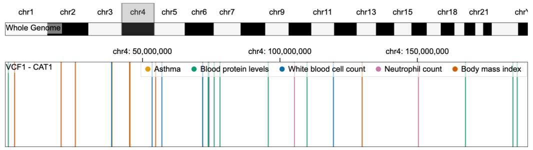	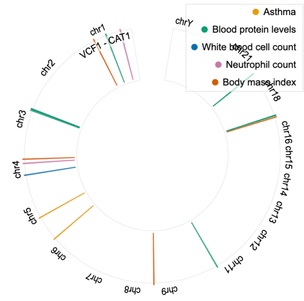
2	#1<br>File Format: BIGWIG<br>Attributes: 1 Quantitative<br>Extent: Point<br>Density: Contiguous<br>Connection: No<br><br>#2<br>File Format: VCF<br>Attributes: 1 Quantitative<br>Extent: Point<br>Density: Sparse<br>Connection: No	Task: In this overview, find the areas in the genome where both quantitative attributes have high values. Do not change the zoom level.	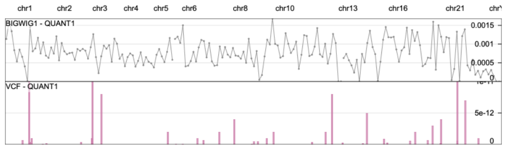	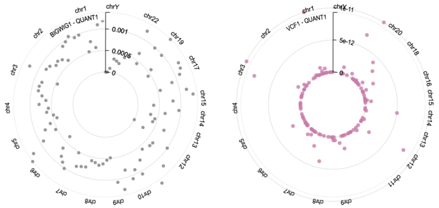
3	#1<br>File Format: BIGWIG<br>Attributes: 1 Quantitative<br>Extent: Point<br>Density: Contiguous<br>Connection: No	Task: Identify if there are any global patterns of interest in the data. Do not change the zoom level.	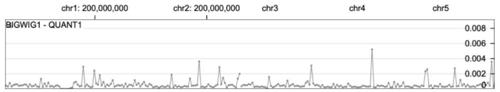	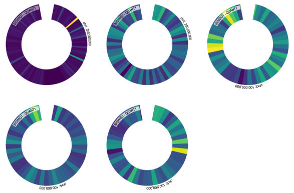

Data, Tasks and Stimuli for Quantitative Evaluation (Study 2) – Page 2/3

Study Web App: https://aditeyapandey.github.io/genorec-study/

Scenario	Data	Task	GenoREC	Alternate
4	#1<br>File Format: BIGWIG<br>Attributes: 1 Quantitative<br>Extent: Point<br>Density: Contiguous<br>Connection: No<br><br>#2<br>File Format: BED<br>Attributes: 1 Categorical<br>Extent: Segment<br>Density: Sparse<br>Connection: No	Task: Navigate to the window: Chr3: 60,000,000 - Chr3: 100,750,000. In this window, find the approximate location of the peak value and characterize the distribution of the categorical variable, i.e., are its values similar or do they vary?	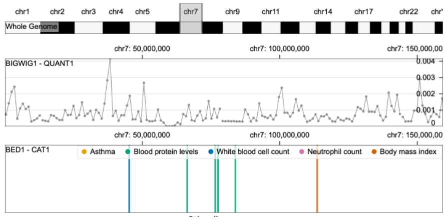	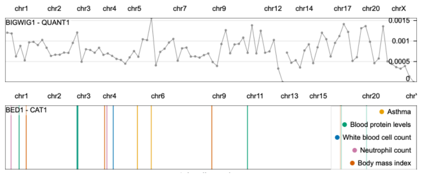
5	#1<br>File Format: BIGWIG<br>Attributes: 1 Quantitative<br>Extent: Point<br>Density: Contiguous<br>Connection: No<br><br>#2<br>File Format: BIGWIG<br>Attributes: 1 Quantitative<br>Extent: Point<br>Density: Contiguous<br>Connection: No	Task: Compare values of the quantitative variable Chr3:100,000,000 and Chr3:150,000,000 with values between Chr6:50,000,000 and Chr6:100,000,000.	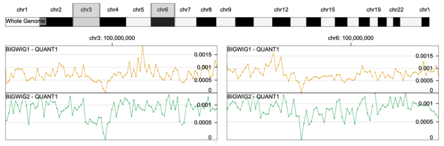	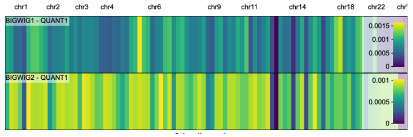
6	#1<br>File Format: BIGWIG<br>Attributes: 1 Quantitative<br>Extent: Point<br>Density: Contiguous<br>Connection: No	Task: Navigate to window Chr6:160,000,000 - Chr6: 165,000,000 and look for the gene "PRKN". Find the second smallest value within the gene body.	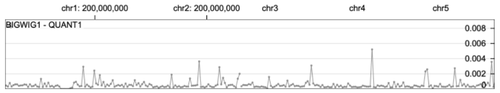	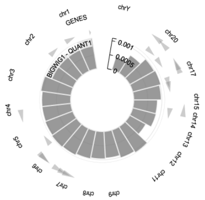

Data, Tasks and Stimuli for Quantitative Evaluation (Study 2) – Page 3/3

Study Web App: https://aditeyapandey.github.io/genorec-study/

Scenario	Data	Task	GenoREC	Alternate
7	#1<br>File Format: BED<br>Attributes: 1 Categorical<br>Extent: Segment<br>Density: Sparse<br>Connection: No<br><br>#2<br>File Format: BED<br>Attributes: 1 Categorical<br>Extent: Segment<br>Density: Sparse<br>Connection: No	Task: Identify regions where both categorical variables have high data density in Chr1 - Chr7.	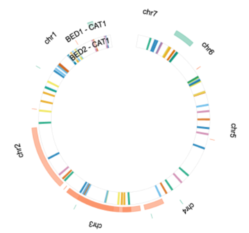	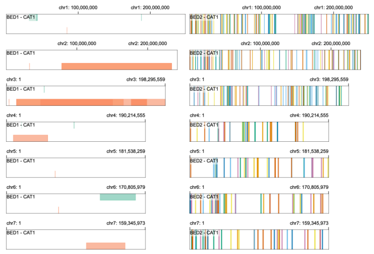
8	#1<br>File Format: BED<br>Attributes: 1 Categorical<br>Extent: Segment<br>Density: Sparse<br>Connection: No<br><br>#2<br>File Format: BED<br>Attributes: 1 Quantitative<br>Extent: Segment<br>Density: Sparse<br>Connection: No<br><br>#3<br>File Format: BED<br>Attributes: 1 Text<br>Extent: Segment<br>Density: Sparse<br>Connection: No	Task: Navigate to window Chr13:48,000,000 - Chr13: 52,000,000. Find the region with a peak and corresponding to the peak value find the category and gene annotation.	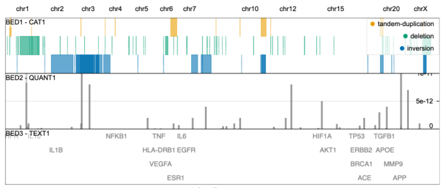	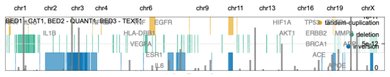
9	#1<br>File Format: BEDPE<br>Attributes: 1 Categorical<br>Extent: Segment<br>Density: Sparse<br>Connection: Yes	Task: Compared to Ch3 does Ch 8 have a lower or higher number of intrachromosomal connections (connections that start and end within the chromsome)?	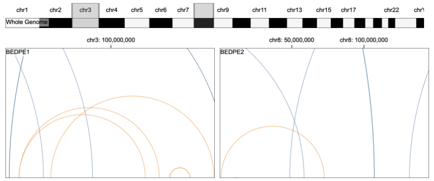	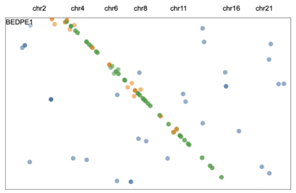
