# Supplementary material for: GenoREC: A Recommendation System for Interactive Genomics Data Visualization
Source: IEEE Trans Vis Comput Graph. Author manuscript; Available in PMC 2023 Apr 5. (PMC10067538; doi:10.1109/TVCG.2022.3209407)
Supplement: Supplementary Material [file NIHMS1846026-supplement-Supplementary_Material.zip › Supplemental Materials GenoREC/Study 2/GenoRec Study 2.pdf]

GenoRec Study

Evaluators: Aditeya Pandey
Email: pandey.ad@northeastern.edu

Information Sheet

Purpose of Study: We will show recommendations from a system and gather your feedback on the system's output.

Duration: The study will be 30 mins long.

Data Privacy: All the information collected from you in this study will be stored in a secure protected location. All user information will be de-identified for any presentation and communication purpose.

Your Rights: Throughout the interview you are free to skip any question or you can leave the interview at any time you wish. You can also ask any question you have at any point in the study.

IRB: This study has received an exemption from Harvard IRB.

If you have any questions about the study you can reach out to: pandey.ad@northeastern.edu, sehi.lyi@hms.harvard.edu or nils@hms.harvard.edu

Personal Information

1. Name*
2. Gender
3. Affiliation (Academic Institute or Company)
4. Years of experience in genomics

What is GenoRec?

GenoRec recommends genomic visualization designs based on data characteristics and analytical tasks.

The system allows users to describe the characteristics of their datasets and uses the descriptions to generate the recommendation.

Applications: GenoRec recommendations can serve as a starting point for genomic analysts to enhance their visualizations or as a final visualization design for analyzing genomics data.

Study Overview

1. You will see a description about one or more genomics data files (e.g., “BED”) and an analysis task (e.g., “compare two regions”).

2. For each description, you will also see two **interactive** visualizations for the given description one at a time.

3. Your task in each session is to rate each visualization whether or not you are **willing to accept the given visualization for the give genomics files and analysis task** (i.e., the description).

4. Your response will be recorded on the scale of 1 to 5 . You are not required to justify your scoring.

5. **If it is taking too long to finish a task, please provide a rating and move forward.**

6. **Both visualizations should be ranked individually.**

Data Representation

1. File Format: VCF
2. Attributes: Quantitative, Categorical, Text
3. Feature Extent: Point or Segment
4. Feature Density: Sparse or Contiguous
5. Network: Yes or No

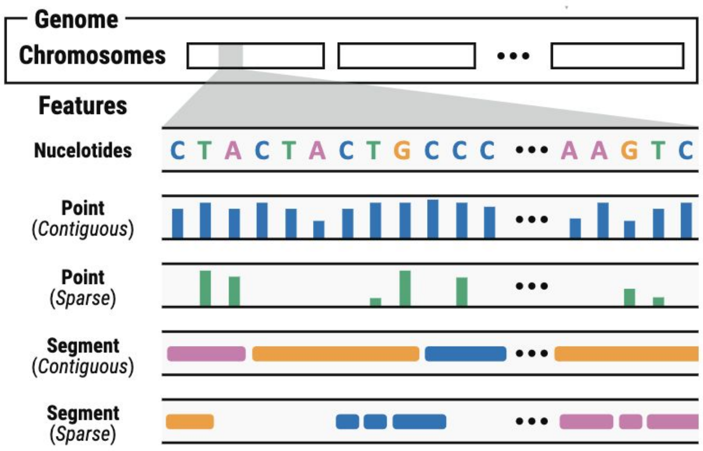

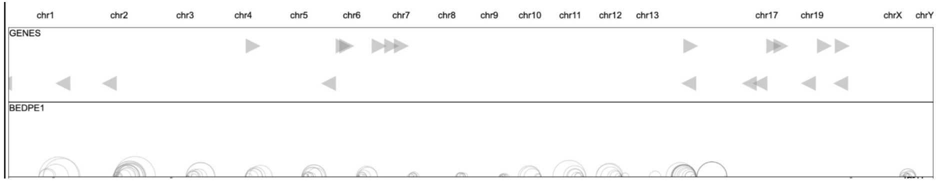

Dataset Representation Example

Chr	POS	Alt
20	14370	A
20	17330	T
:	:	:
:	:	:

VCF

File Format: VCF
Attributes: 1 Categorical
Extent: Point
Density: Sparse
Connection: No

Study Data Description

Task Options

1. **Identify and analyze a region of interest**
   a. Example: Navigate to gene A and check its functional annotation.
2. **Compare two regions of interest**
   a. Example: Compare the gene expression level between gene A and gene B.
3. **Overview of the entire genome**
   a. Example: Identify genes where the feature expression levels are low or high.

Any Questions?

https://aditeyapandey.github.io/genorec-study/
