# Supplementary material for: GenoREC: A Recommendation System for Interactive Genomics Data Visualization
Source: IEEE Trans Vis Comput Graph. Author manuscript; Available in PMC 2023 Apr 5. (PMC10067538; doi:10.1109/TVCG.2022.3209407)
Supplement: Supplementary Material [file NIHMS1846026-supplement-Supplementary_Material.zip › Supplemental Materials GenoREC/Study 2/Appendix for Study 2.docx]

### Study 2

**Participants:** The participants spanned occupations across industry and academia: three participants work in industry with biotech companies, two participants are researchers at genomics research institutes, and eight participants are from academia (one data scientist, one post-doc, four PhD Students, and two Master's Students). The diversity in occupational and range of experience was on purpose to ensure an appropriate sample population. For the analysis of sample size, we used alpha = 0.5 and power=0.8.. Additionally, we simulated a range of median utility difference between GenoREC's top-ranked recommendation and alternate recommendation and computed a range of estimated sample sizes using ``G*Power". Taking a median of all samples, we predicted a sample size of twelve participants for the study.
